# Prevalence and characteristics of breakthrough cancer pain in an outpatient clinic in a Catalan teaching hospital: incorporation of the Edmonton Classification System for Cancer pain into the diagnostic algorithm

**DOI:** 10.1186/s12904-018-0336-y

**Published:** 2018-05-28

**Authors:** Jaume Canal-Sotelo, Javier Trujillano-Cabello, Philip Larkin, Núria Arraràs-Torrelles, Ramona González-Rubió, Mariona Rocaspana-Garcia, Eva Barallat-Gimeno

**Affiliations:** 1 Hospital Universitari Arnau de Vilanova, UFISS GSS, Alcalde Rovira Roure, 80, 25198 Lleida, Spain; 2 Faculty of Medicine, Universitat de Lleida, Montserrat Roig 2, 25198 Lleida, Spain; 3 University College Dublin, School of Nursing and Midwifery and health Systems Health Sciences, Belfield, Dublin, Ireland; 4 Hospital Universitari Santa Maria, Alcalde Rovira Roure, 44, 25198 Lleida, Spain; 5 Hospital Universitari Santa Maria, Alcalde Rovira Roure, 44, 25198 Lleida, Spain; 6 Faculty of Nursing and Phisiotherapy, Universitat de Lleida, Montserrat Roig 2, 25198 Lleida, Spain

**Keywords:** Breakthrough cancer pain, Palliative care, ECS-CP, Neuropathic pain, Addictive behaviour, Psychological distress

## Abstract

**Background:**

Breakthrough cancer pain (BTcP) is defined according to its principal characteristics: high intensity, short time interval between onset and peak intensity, short duration, potential recurrence over 24 h and non-responsiveness to standard analgesic regimes. The Edmonton Classification System for Cancer Pain (ECS-CP) is a classification tool that evaluates different dimensions of pain.

The aim of this study was to measure prevalence and the main characteristics of BTcP in a sample of advanced cancer patients and to explore the complexity observed when ECS-CP is incorporated into BTcP diagnostics algorithm.

**Methods:**

Descriptive prevalence study (Retrospective chart review). Davies’ algorithm was used to identify BTcP and ECS-CP was used to recognize appropriate dimensions of pain. The study was conducted in a sample of advanced cancer patients attending hospital outpatient clinic in Lleida, Spain. 277 patients were included from 01/01/2014 to 31/12/2015. No direct contact was made with participants. The following information was extracted from the palliative care outpatient clinic database: age, gender, civil status, cognitive impairment status, functional performance status and variables related to tumour. Only BTcP cases were included.

**Results:**

Prevalence of BTcP was 39.34% (63.9% men). Mean of age was 68.2 years. Main diagnosis was lung cancer (*n* = 154; 31.6%). Metastases were diagnosed in 83% of the sample. 138 patients (49.8%) were diagnosed with 1 type of BTcP and 139 (50.2%) were diagnosed with more than one type of BTcP. In total, 488 different types of BTcP were recorded (mean 1.75 ± 0, 9), 244 of these types (50%) presented a component of neuropathic pain. Addictive behaviour, measured through CAGE test, was present in 29.2% (*N* = 81) of the patients and psychological distress was present in 40.8% (*n* = 113).

**Conclusions:**

Prevalence of BTcP (39.34%) is similar to the one reflected in the existing literature. Study results indicate that the routine use of ECS-CP in a clinical setting allows us to detect more than one type of BTcP as well as additional complexity associated with pain (neuropathic, addictive behavior and psychological distress).

## Background

In 1989, Portenoy and Hagen [1] defined breakthrough cancer pain (BTcP) as the transient exacerbation of pain occurring in a patient with otherwise stable pain in receipt of chronic opioid therapy. This pain is one of the most difficult pain syndromes to treat. The term encompasses a diverse group of transient pains that vary in their relationship to the fixed analgesic dose, temporal characteristics, precipitating events, predictability, pathophysiology, and aetiology [1, 2].

Later, BTcP was redefined as a transient exacerbation of pain that occurs either spontaneously, or in relation to a specific predictable or unpredictable trigger, despite relatively stable and adequately controlled background pain [3]. More recently, other authors [4–7] have improved BTcP definition by adding severity of intensity and the length between 30 and 60 min.

Prevalence of BTcP varies between 19 and 95% [8–11]. This is explained by the different definitions found in the literature and also depending on the area where the data are collected (inpatient or outpatient patients).

The prevalence of BTcP assessed in outpatient clinics is 39.9% and in those assessed in palliative care units, is 80.5% [12].

It is therefore difficult to diagnose BTcP. For this reason, many Scientific Societies related to cancer, palliative care and pain, work to clarify the definition and the accurate diagnosis of BTcP [13–17].

In the same way, different instruments have been defined to facilitate the diagnostic approach of BTcP. We highlight the Alberta Breakthrough Pain Assessment Tool for Cancer Patients [18], the breakthrough pain assessment tool (BAT) in cancer patients [19] and the Italian Questionnaire for Breakthrough Pain (IQ-BTP) [20].

To improve the sensitivity of the diagnosis of BTcP, several authors developed the so-called “Davies algorithm” [21], recently validated by Weber K et al. [22]. Although the use of this algorithm is widespread, it is not designed to replace clinical assessment.

Literature refers to BTcP as a single clinical entity with several possible episodes in a single patient and the fact that more than one different types of BTcP with several episodes each in the same patient has not yet been explored [16, 23, 24].

The pharmacological treatment of BTcP is based on the use of the three-step ladder of the WHO [25, 26]. Considering the characteristics of BTcP in terms of temporality and intensity, only Rapid Onset Opioids (ROOs), mainly fentanyl, have been shown to be effective [27–29]. Zeppetella and Davies [30] conclude that both oral and intranasal-Trans-mucosal fentanyl are effective for the treatment of BTcP episodes.

Early pharmacological approach is the cornerstone of the treatment of BTcP [31]. Its improvement will also enhance both the quality of life and the functionalism of the patient [32].

The first choice of treatment is always oral and in many cases, for the treatment of BTcP, the choice is fast bioavailability treatments such as fentanyl. The rapid bioavailability of fentanyl-based ROOs may lead to episodes of abuse of these drugs; therefore it is advisable to minimize the risk with a detailed appropriately assessment [33, 34].

The Edmonton Classification System for Cancer Pain (ECS-CP) derives from the Revised Edmonton Staging System (rESS) from which construct, inter-rater reliability, and predictive validity evidence have contributed to the development of the ECS-CP. The five features of cancer pain included -Pain Mechanism (N), Incident Pain (I), Psychological Distress (P), Addictive Behavior (A) and Cognitive Function (C)- have demonstrated value in predicting pain management complexity [35, 36].

The ECS-CP is a clinically relevant systematic framework, which is able to detect differences in salient pain classification features across diverse settings and countries [37].

It is known that BTcP is difficult to diagnose and to treat. We hypothesized that if we add the ECS-CP during the diagnosis process, we find an added complexity together with the incident features of the cancer pain. This is because we can find other characteristics of pain such as the neuropathic component, addiction and psychological discomfort.

Therefore, the objectives of this retrospective review were:

1. To describe the characteristics of the population studied and the prevalence of BTcP in a sample of advanced cancer patients treated at an outpatient clinic.
2. To determine the number of different types of BTcP diagnosed in each patient, regardless the number of episodes of BTcP.
3. To explore the different pain features associated to the diagnosis of BTcP

## Methods

This was a retrospective and anonymous database review of the patients attending for the first time at Palliative Care outpatient clinic, which is maintained at the Lleida University Hospital in Catalonia (Spain).

This study was approved by the Ethics Committee of the Hospital Universitari Arnau de Vilanova in Lleida. All data-analysis was performed anonymously without an additional informed consent, according to its recommendations. The administrative permissions required were obtained in order to review patient records and use the data.

This Palliative Care outpatient clinic attends advanced cancer patients early in the disease course as well as patients that are not receiving active treatment. The patients were referred to the clinic by their reference oncologist and the palliative care consultation team was the responsible organ of the pain management.

Inclusion criteria: age > 18 years, diagnosis of advanced cancer (non haematological) assisted at the outpatient clinic of the Palliative care outpatient clinic suffering, from BTcP due to cancer and without any cognitive impairment (Pfeiffer test ≥ 4 errors). End-of-dose pain was specifically excluded.

We defined BTcP according to Boceta et al. [38] as a transitory exacerbation of pain lasting less than 60 min, which occurs spontaneously or in association with a specific predictable or unpredictable trigger at some point during the day in cancer patients, despite relatively and adequately controlled background pain. BTcP was considered as those that have different characteristics in terms of localization, intensity, mechanisms that trigger it or intrinsic characteristics of pain (neuropathic vs. nociceptive). The same type of BTcP can present with several episodes (maximum 4 episodes per day). The literature review shows that there is not a broad consensus about definition of BTcP therefore, to facilitate the methodology of data collection; we included equal terms BTcP and incidental pain.

A physician, also responsible for assessing pain and other symptoms, evaluated all the patients attended in the outpatient clinic. Pain was assessed using a Visual Analogic Scale (VAS) and the cut-off value (VAS scale) of patients for their background pain was VAS ≤ 3 during the previous 7 days. All patients who reported adequately controlled background pain (VAS ≤ 3) in the previous week were further evaluated exhaustively. The procedure to diagnose BTcP was done following the algorithm of Davies [21] and according to the consensus recommendations from the Spanish Pain Society. The algorithm indicates that baseline pain must be adequately controlled before a diagnosis of BTcP can be considered. Each BTcP were located anatomically in the painful area and each patient could present different types of pain. For each type of BTcP, the ECS-CP test was later applied to assess additional complexity. The ECS-CP classifies the different pain features according to its origin. This way, the neuropathic and incident component of pain can be secondary to the tumor itself while the psychological discomfort and addictive behaviour can be considered personality traits. Therefore, the analysis of both the neuropathic (N) and the incident (I) component of pain was done over the total number of different types of BTcP detected and the Psychological (P) and addictive (A) traits were analysed over the total number of patients included. The Cognitive (C) component was specifically excluded.

This is the usual protocol applied in order to study pain when a patient is assessed first time at the outpatient clinic.

### Data collection

The following information was extracted from the chart review: age, gender, civil status, cognitive status measured with Pfeiffer test [39, 40], functional performance status measured with Barthel test [41, 42] and with Palliative Performance Scale version 2 test (PPSv2) [43, 44]. Variables related to the tumour were obtained (primary tumour diagnosis, metastatic disease and locally advanced disease).

We also extracted the information related to BTcP as following:

- Related Factors: predictable, unpredictable or idiopathic (volitional, non-volitional or idiopathic).
- Cause of pain: tumour, treatments received or idiopathic cause.
- Intensity of pain: measured through a VAS scale. Minimum and maximum intensity were recorded. The difference between VAS minimum intensity (VAS min) and VAS maximum intensity (VAS max) should be ≥ 3 points measured with scale from 0 to 10.

The ECS-CP was applied in order to detect additional pain features other than incident pain in the same patient. The neuropathic component of pain was assessed through the Doleur Neuropathique-4 questionnaire (DN4) [45, 46] altogether with the clinical examination. For the psychological distress we followed the Clinical Practice Guidelines in Oncology (NCCN) and a VAS ≥ 4 in either anxiety or depression was the cut-off point [47]. Regarding the addictive behaviour only the addiction to alcohol was collected and measured through Cut down, Annoyed, Guilty and Eye-opener questionnaire (CAGE) [48]. Two or more “yes” responses indicated the possibility of alcoholism.

For each pain features detected by the ECS-CP (NIPAC), one point was given.

Even if the number of BTcP episodes were specifically registered in the patients files, they were not included in the data analysis.

### Data analysis

The data included all patients attending the outpatient clinic of the Catalan University Hospital Arnau de Vilanova of Lleida between 2014 and 2015. The information for the study was extracted between June and October 2016.

The study was carried out in two separate phases; in the first phase the data analyzed was related to the total number of patients with pain included consecutively in the study and this sample was further studied according to the number of different types of pain found (1 types vs. > 1 type of pain). In the second phase, data analyzed was related to the total number of different types of BTcP individualized after having used the ECS-CP in the diagnostic algorithm and the sample was also further analyzed depending of the pain intensity. We considered mild and moderate pain if the VAS < 7 and severe pain included VAS ≥ 7.

Statistical analyses were conducted using SPSS Statistics 20 (IBM Corporation) and Microsoft Excel (Microsoft Corporation) software [49]. Continuous variables were summarized as means and standard deviations (SD). Categorical variables were summarized as percentages (absolute numbers). Univariate analysis was performed using the Wilcoxon or Chi square test without correction for continuity for comparison among groups of continuous and categorical variables, respectively. Statistical significance was assumed at a 0.05 level (*P* < 0.05).

## Results

**Fig. 1:**
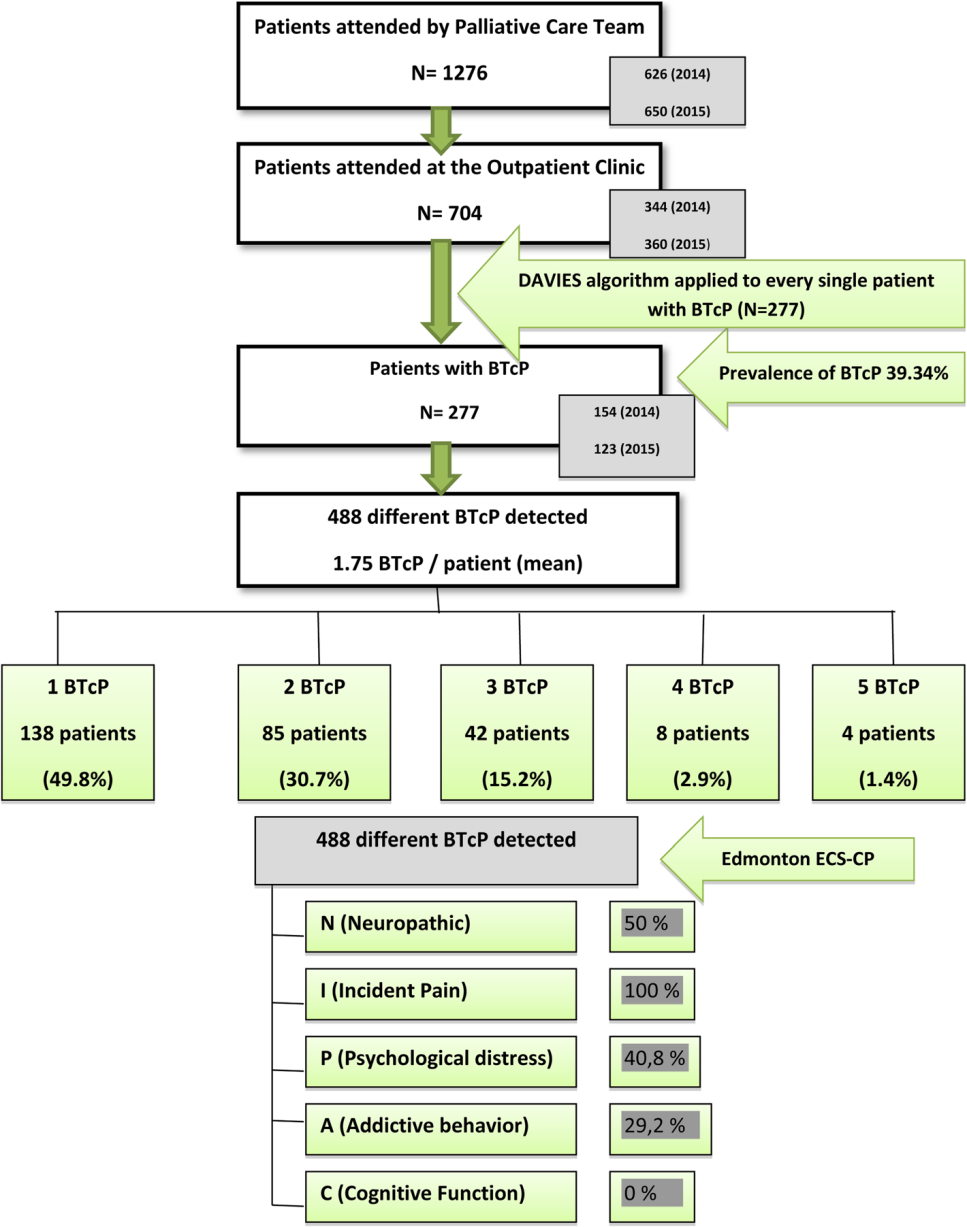
Flow Chart of BTcP diagnosis process. Flow chart of patients visited first time in the Palliative care outpatient clinic. Prevalence of BTcP. Patients diagnosed with BTcP and different types of BTcP according to the ECS-CP classification

The palliative care team visited a total of 1276 patients for the first time at the University Hospital Arnau de Vilanova in Lleida, Catalonia (Spain) between January 2014 and December 2015. 704 of them (55.17%) attended the outpatient clinic. 303 patients had pain, and 277 were diagnosed of BTcP and included to the chart review study. Mean age was 68.2 ± 13 years while men accounted for 67.9% of the sample. Lung cancer (31%) was the most prevalent cancer diagnosis and metastatic disease was found in 83% of the sample. A prevalence of 39.34% of BTcP (277/704 patients) was found*.* A total of 488 different types of BTcP were detected (mean of 1.75 ± 0.9 types of BTcP per patient). Up to 5 different types of BTcP were found among the patients and 50,2% of patients (*N* = 139) accounted for ≥ 2 types of BTcP (Fig. 1).

Main characteristics of the population studied are showed in Table 1*.* Addictive behavior was detected in 29.2% of the sample and the psychological discomfort was detected in 40.8%. This table also shows the results according to two groups of patients (1 type vs > 1 type). The group of patients with > 1 type was younger (66 ± 12, *p = 0.002),* had more metastatic disease (90.6%, *p = 0.001*) and presented with more psychological discomfort (47.5%, *p =* 0.023). Patients with 1 type of BTcP presented addictive behavior (CAGE) (34.8%, *p* = 0.043).

**Table 1:** Sociodemographic and medical characteristics of the patients (*N* = 277) in relation to the number of BTcP

	Total (*n* = 277)	BTcP		*p* ^*b*^
		BTcP (1 type) *N* = 138	BTcP (> 1 type) *N* = 139	
Age (years)^a^	68.2 ± 13	70.4 ± 13	66 ± 12	0.002
Gender (men) (%)	67.9	73.2	62.6	0.059
Civil Status (%)				0.854
Married/couple	71.1	69.6	72.7	
Single	6.5	5.8	7.2	
Separated/divorced	7.6	8	7.2	
Widowed	10.8	11.6	10.1	
Missing	4	5.1	2.9	
Type tumor (%)				0.508
Lung	31	31.2	30.9	
Upper digestive	19.9	21	18.7	
Lower digestive	19.1	18.8	19.4	
Ear-Nose and Throat	8.7	10	7.2	
Genitourinary male	8.7	6.5	10.8	
Genitourinary female	4	2.2	5.8	
Other	8.7	10.1	7.2	
Metastatic disease (%)	83	75.4	90.6	0.001
Pfeiffer Test^a^	0.8 ± 1	0.9 ± 1	0.6 ± 1	0.068
Barthel Test^a^	87 ± 17	87 ± 18	87 ± 15	0.409
PPSv2 Test^a^	64 ± 12	64 ± 12	63 ± 11	0.131
Pain features				
Addictive	29.2	34.8	23.7	0.043
Psychological	40.8	34.1	47.5	0.023
Number of BTcP types^a^	1.75 ± 0.9			

^a^mean ± standard deviation

^b^Comparison between groups with the χ^2^ test and for continuous variables with the Mann-Whitney test

In Table 2, the analysis was performed taking into account the number of different types of BTcP detected (*N* = 488). The use of the ECS-CP tool on each type of BTcP allowed us to detect that, together with the incident feature of pain, 50% (*N* = 244) had a neuropathic component. Non-volitional component of BTcP was detected in the 63.7% of the sample. The sum of the different pain features detected by the ECS-CP (NIPAC) when applied on the sample of 488 different types of BTcP is 2.2 ± 1.

**Table 2:** Characteristics of episodes of incidental pain (*n* = 488) according to intensity (maximum VAS ≥ 7)

	SAMPLE (*n* = 488)	VAS MAX < 7 (*n* = 305)	VAS MAX ≥ 7 (*n* = 183)	*p* ^*b*^
Due to Tumour	93,0	91,8	95,1	*0,168*
Due to treatment	8,0	8,5	7,1	*0,575*
Volitional	36,3	36,7	35,5	*0,789*
Non volitional	63,7	64,1	35,9	*0,618*
Neuropathic condition	50,0	45,4	63,0	*0,001*
∑NIPAC	2,2 ± 1	2,2 ± 1	2,3 ± 1	*0,044*
Type tumor				*0,007*
Lung	31,6	35,4	25,1	
Upper digestive	18,0	18,7	16,9	
Lower digestive	20,1	20,3	19,7	
ENT	7,6	6,9	8,7	
Genitourinary male	8,4	8,9	7,7	
Genitourinary female	5,1	4,3	6,7	
Other	9,2	5,6	15,3	

Values as percentage

^a^mean ± standard deviation

^b^Comparison between groups with the de χ^2^ test and for continuous variables with the Mann-Whitney test

## Discussion

This retrospective study was designed to determine several outcomes related to BTcP in a sample of advanced cancer patients who attended the outpatient clinic of a University hospital during a two-year period (2014–2015).

We identified a prevalence of 39.34% of BTcP in the sample of patients screened for the study. The application of Davies algorithm and a close clinical examination were the cornerstone for defining pain. This result is consistent with Deandrea et al. [12] who after a bibliographic research, stated a prevalence of BTcP of 39.9% for cancer patients attended at the outpatient clinic. Similar outcome data are reported by Margarit et al. [50] who, after reviewing data from the *American Pain Foundation*, show a prevalence of 35% for those cancer patients seen on an ambulatory regime.

To our knowledge, this study provides the first data regarding the fact that a single patient can present with more than one type of BTcP. Previous studies only address this subject as a single patient having different episodes of the same BTcP. On the current study, we found that a total of 488 different types of BTcP were assessed in a sample of 277 patients. Each patient had an average of 1.75 BTcP. We remark that more than half of the patients (139/277) were found to report more than one type of BTcP. Up to 12 patients presented with 4 or 5 different types of BTcP (4.3%) while 127 patients (45.9%) presented with 2 or 3 different types of BTcP.

Younger patients and those presenting with metastatic disease variables were found statistically significant.

The classification ECS-CP provides further insight into several characteristics of pain like neuropathic, psychological distress and addictive behavior features. As the Incident component of pain is already included in the ECS-CP, all types of pain found in our study had a BTcP component. The addictive behaviour (A) was sensibly higher (29.2%) than the found in the literature. Parsons et al. [51] and Dev et al. [52] found in their studies a prevalence of addictive behaviour of 17% in cancer patients attending an outpatient clinic while Chow et al. [53] found a poor 7% prevalence rate in an outpatient palliative radiotherapy clinic. Even if all studies screened the addictive behaviour using the CAGE questionnaire, differences found can be explained by the fact that we recorded the CAGE in current or former drinkers.

The sample of 488 different types of pain found was further divided according to pain intensity. Only neuropathic nature of pain, the summation of the ECS-CP and the type of cancer showed significant statistical differences.

The ECS-CP has demonstrated its utility in routine clinical practice. Arthur et al. [54] found that neuropathic pain and psychological distress were associated with higher pain intensity. Also a higher sum of ECS-CP features was associated with higher pain intensity. More recently the same author found that increasing sum of ECS-CP features was not predictive of pain management complexity. Our study shows that a higher sum of ECS-CP features was found among the group of types of BTcP with a VAS intensity ≥ 7.

However the study has limitations; first, the study was carried out in a single institution and data available from medical records were recorded by a single physician during clinical interviews. Second, data from addiction behaviour only included alcohol screening through the CAGE questionnaire.

Our study did not aim to analyse any specific pharmacological treatment the patients received. Our study shows a high prevalence of the neuropathic component of BTcP, further studies have to address this finding.

The strengths and limitations of this study are the following:

- This is a retrospective study and prevalence rates are reported from a single institution and a single physician recorded the data available from medical records during clinical interviews.
- This study includes BTcP and incidental pain in equal terms.
- Data from addiction behaviour only included alcohol screening through the CAGE questionnaire.
- This study included a large cohort of patients who had BTcP.
- This study supports the hypothesis that a single patient can present more than one type of BTcP.
- This study supports the use of screening tools to better categorize diagnose of Cancer pain.

## Conclusions

Our study provides data regarding prevalence of BTcP in a palliative care outpatient clinic in a single Teaching Hospital and the result of 39.94% is similar to those found in the literature.

This retrospective chart review allows determining the number of different types of BTcP diagnosed in each patient, regardless the number of episodes of BTcP. This is possible with the routine use of the ECS-CP in tandem with the Algorithm of Davies when exploring BTcP in cancer patients.

This study explores the different pain features associated to the diagnosis of BTcP. Clinicians have to take into account several pain features such as the neuropathic nature of pain, psychological distress and addictive behaviour, as the optimal therapeutic approach can change.

The existence of more than one type of BTcP in each patient adds more complexity to the pain assessment.

## Abbreviations

A: Addictive Behavior
BAT: Breakthrough pain assessment tool
BTcP: Breakthrough Cancer Pain
C: Cognitive Function
CAGE: Cut down, annoyed, guilty and eye-opener questionnaire
DN4: Doleur neuropathique-4 questionnaire
ECS-CP: Edmonton Classification System for Cancer Pain
ENT: Ear, Nose and Throat
I: Incident Pain
IQ-BTP: Italian Questionnaire for Breakthrough Pain
N: Pain Mechanism
NCCN: Clinical Practice Guidelines in Oncology
P: Psychological Distress
PPSv2: Palliative Performance Scale version 2 test
rESS: Revised Edmonton Staging System
ROOs: Rapid Onset Opioids
SD: standard deviations
VAS max: VAS maximum intensity
VAS min: VAS minimum intensity
VAS: Visual analogic Scale
